# Temperature during conidiophore development primes *Aspergillus fumigatus* spore transcriptome for asexual or sexual reproduction

**DOI:** 10.1128/mbio.01831-26

**Published:** 2026-09-01

**Authors:** Justina M. Stanislaw, Michelle Momany

**Affiliations:** 1 Fungal Biology Group and Plant Biology Department, The University of Georgia 1355, Athens, Georgia, USA; Instituto Carlos Chagas, Curitiba, Brazil

**Keywords:** conidiation, sporulation, dormancy, *Aspergillus*, development

## Abstract

*Aspergillus fumigatus* is a thermotolerant saprobe found in soils and plant debris worldwide and an important pathogen of humans, causing two million deaths annually. *A. fumigatus* makes abundant asexual spores (conidia), which are widely distributed by wind and can be inhaled from the environment. In susceptible individuals, inhaled conidia break dormancy, germinate, and grow in the lung, leading to serious disease. Recent work has shown that conidia made at 37°C and 50°C have different morphologies and germination kinetics. While the asexual cycle is well-characterized at 37°C, much less is known about the asexual cycle at 50°C. Here, we combine flow cytometry and transcriptomics to track morphology and gene expression in the hyphae, conidiophores, and conidia of *A. fumigatus* during asexual development at 37°C or 50°C. We show that the temperature during a narrow time window in late-stage conidiophore development dictates resulting conidial morphology, transcriptional program, and germination kinetics. As expected, conidiation at 37°C resulted in upregulation of *brlA*, encoding the master regulator of asexual development, and its downstream targets in conidiophores and conidia. Surprisingly, conidiation at 50°C resulted in upregulation of *MAT1-1-1*, encoding the master regulator of sexual development, and its downstream targets in conidiophores and conidia. Our findings suggest that temperature during late conidiophore development transcriptionally primes conidia for asexual, parasexual, or sexual development, enhancing chances of survival for progeny. Our findings are especially relevant for agricultural compost, where a wide gradient of temperatures exists, abundant *A. fumigatus* has been isolated, and resistance to antifungals is thought to evolve.

**IMPORTANCE:**

The human pathogen *Aspergillus fumigatus* has been found in natural and agricultural environments around the world. Disease is acquired when susceptible individuals inhale airborne asexual spores from the environment, which in agriculture generally includes proximity to compost and plant debris piles. This work shows that the environmental temperature when *A. fumigatus* spores are made determines the transcriptomes of those spores, priming them for future asexual or sexual development. The survival of asexual and sexual spores is very different at different temperatures, so these results are important for understanding how this pathogen survives in varied hostile environments. In addition, there are very few antifungal drugs with which to treat *A. fumigatus* infections, and resistance is increasing, driven in part by agricultural use of fungicides. These results suggest that higher temperatures during asexual spore formation can lead to increased sexual reproduction and greater chances to evolve antifungal resistance.

## INTRODUCTION

The filamentous fungus *Aspergillus fumigatus*, a World Health Organization-designated fungal critical priority pathogen (1), is estimated to kill 2 million people annually (2). *A. fumigatus* is found in soil and leaf litter across varied environments and makes abundant dormant asexual spores (conidia) that are dispersed by wind. *A. fumigatus* disease is initiated when conidia are inhaled from the environment by a susceptible individual, break dormancy, and grow in the lungs. Although *A. fumigatus* primarily reproduces asexually, recent findings show that sexual reproduction also occurs in nature, where it produces sexual spores (ascospores) that are important for generating the phenotypic diversity that allows *A. fumigatus* to survive in differing environments and to evolve antifungal resistance (3).

**Fig 1:**
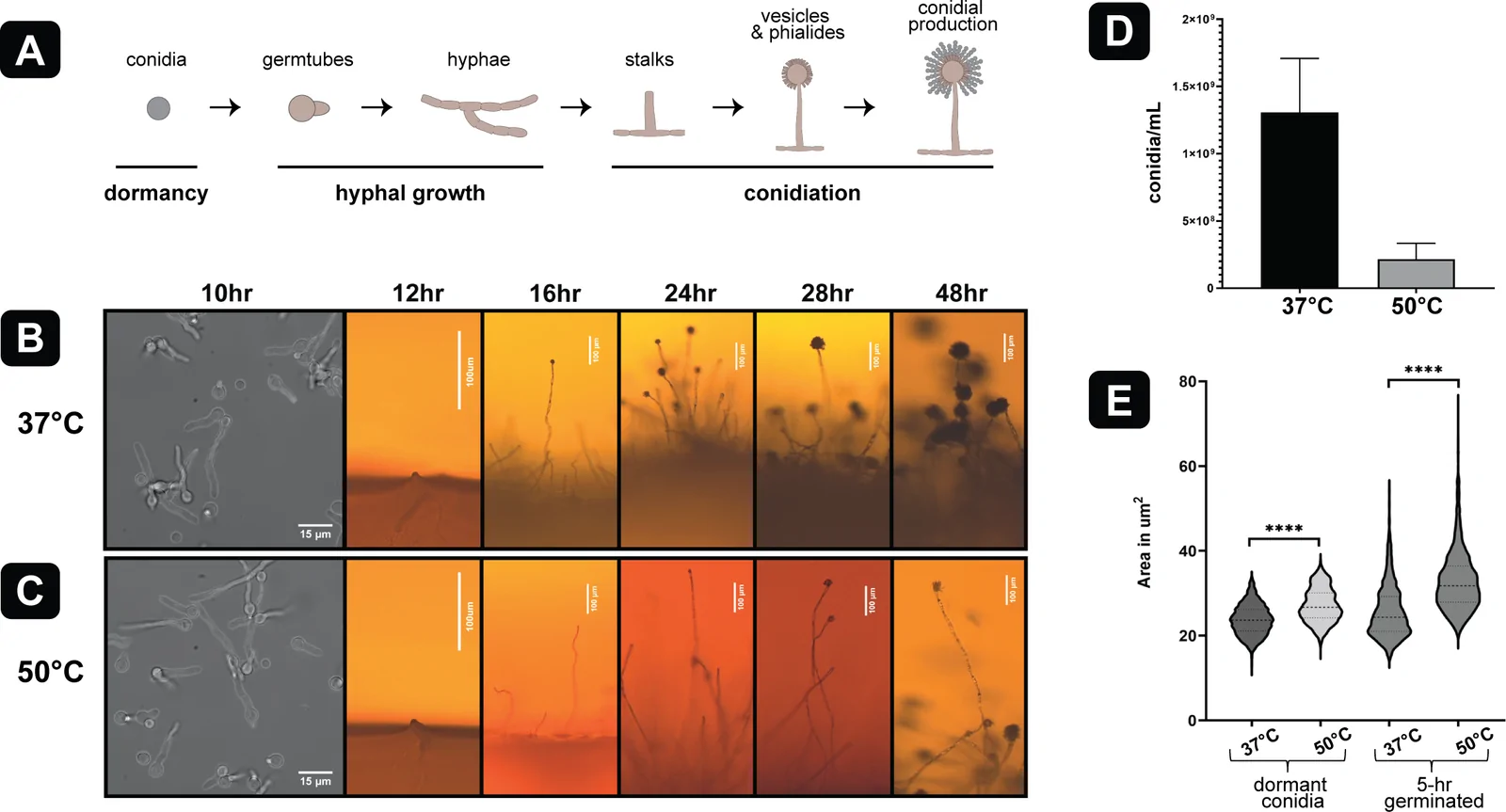
Temperature impacts conidiophore and conidial morphology. (**A**) Key milestones of *A. fumigatus* asexual lifecycle. (**B and C**) Asexual development at 37°C (**B**) or 50°C (**C**). Left column: hyphae 10 h after inoculation. Scale bar = 15 µm. Right five columns: 12–48 h after inoculation. Scale bars = 100 µm. (**D**) Bar graph shows average number of conidia produced for 4 days at 37°C or 50°C from a single petri plate (*n* = 3 biological replicates). (**E**) ImageStream flow cytometry measurements of conidia produced by incubation at 37°C or 50°C for 3 days (dormant) and after 5 h of incubation at 37°C in liquid AMM (germinated). Conidial area in µm^2^ (*y*-axis) with the middle line showing the median. Two-tailed *t*-tests determined statistical significance (Mann-Whitney, nonparametric) using GraphPad Prism version 10. *P* values are indicated by stars: ****P < 0.0001. *n* = 3 of 1,500–3,000 per sample.

In asexual development, the asexual reproductive structure (conidiophore) produces chains of asexual spores (conidia) that contain genetically identical nuclei made by mitosis. Conidia are dormant and contain a single cell cycle-arrested nucleus. When exposed to carbon and water, conidia break dormancy, swell, and eventually resume nuclear division and extend germ tubes that become hyphae during vegetative growth. At the start of asexual development, *A. fumigatus* vegetative hyphae become partitioned by thick cross walls that form foot cells. Aerial hyphae emerge from foot cells and extend apically, forming conidiophore stalks. After a period of extension, stalks round at their tips to develop vesicles, and phialides emerge from the vesicles. Each phialide produces a chain of uninucleate conidia (Fig. 1A) (4). Genetic regulation of asexual reproduction leading to conidia is well-defined at 37°C in standard laboratory conditions, where it is known to be transcriptionally driven by a central regulatory pathway initiated by master regulator BrlA. BrlA binds to and activates expression of genes that contain BrlA response elements (BREs) (5, 6). Multiple direct and indirect downstream targets of BrlA play critical roles in conidial dormancy and maintaining conidial viability (7), including WetA and velvet regulators VeA, VosA, and VelB (6, 8). Conidiation and secondary metabolism are also tightly linked, with BrlA controlling expression of biosynthetic gene clusters (BGCs) leading to the production of specific secondary metabolites during conidiophore development (9–12).

Conidia are abundant and easily dispersed, but they are less genetically diverse than spores derived from other reproductive strategies. Many *Aspergillus* species use parasexual and sexual reproduction, which increase diversity and chances of adapting to varied environments (13, 14). Before two unique fungal hyphae fuse (anastomose) and exchange nuclei, they must overcome barriers that prevent nonself fusion. For many filamentous fungi, including *A. fumigatus,* successful anastomosis requires that partners have specific *het* (heterokaryon incompatibility) genes at several loci, ensuring that only closely related individuals fuse (15, 16). Like asexual development, parasexual development produces chains of conidia on conidiophores by mitosis, with the major difference being that parasex can give rise to variation by chromosome loss and mitotic recombination. Sexual reproduction gives much higher levels of variation via meiosis and meiotic recombination than either asexual or parasexual reproduction. Heterothallic fungi like *A. fumigatus* require partners of opposite mating types for sexual development (*MAT1-1* and *MAT1-2* in *A. fumigatus*). Mating partners secrete pheromones that are recognized by receptors in the opposite mating type partner and allow bypass of heterokaryon incompatibility so that unrelated individuals can fuse. In sexual development, ascospores are made within specialized structures (cleistothecia) by meiosis.

Genetic regulation of sexual reproduction leading to ascospores is also well-defined. Since *A. fumigatus* is heterothallic, strains can only cross if they have opposite mating type idiomorphs *MAT1-1* and *MAT1-2* that harbor the *MAT1-1-1* and *MAT1-2-1* genes, respectively. These mating type genes encode master regulators of sexual development that bind to downstream targets containing the corresponding DNA-binding motifs. MAT1-1-1 and MAT1-2-1 targets directly or indirectly stimulate sexual development, secondary metabolism, and trigger pheromone secretion in the opposite mating type partner to initiate cleistothecia formation and ascospore production (17–21). Although the optimal niche for ascospore formation and germination in nature is not established, it is known that sexual spores thrive at high temperatures and require heat shocks of 65–85°C in order to efficiently break dormancy (22–24).

In recent work, we found that conidia made at 50°C are larger and germinate faster than those made at 37°C (25). Other findings showed that conidial phenotypes and transcriptional profiles are influenced by the growth environment during the asexual lifecycle (26–29). Importantly, larger 50°C conidia have distinct cell wall compositions (28, 29) and are more virulent than those made at 37°C (25). Although it has been established that distinct environments produce distinct conidial morphologies and transcriptomes, it is not known when this variation is determined during the asexual lifecycle of *A. fumigatus*. Here, we combine ImageStream flow cytometry and transcriptomics to investigate the impact of environmental temperature on landmark intermediates in the *A. fumigatus* asexual lifecycle.

We show that environmental temperature during late-stage conidiophore development dictates the resulting conidial phenotype and that once established, the phenotype cannot be altered. We found that transcriptomes of conidiophores made at 37°C are significantly upregulated in *brlA*, which encodes the master regulator of asexual development, and that conidia produced from these conidiophores are upregulated in downstream targets of BrlA. Surprisingly, we also found that conidiophores made at 50°C are significantly upregulated in *MAT1-1-1*, which encodes the master regulator of sexual development, and that conidia produced from these conidiophores are upregulated in downstream targets of MAT1-1-1. Our findings suggest that the growth environment during late-stage conidiophore development directs distinct regulatory pathways that prime the resulting conidia for higher fitness and impact genetic variability.

## RESULTS

### Asexual development at 37°C vs 50°C produces distinct and trackable phenotypes

To determine whether key landmarks of the asexual lifecycle differ at 37°C and 50°C, we inoculated *A. fumigatus* CEA10 (*MAT1-1*) (30, 31) conidia to plates as an evenly spread lawn and incubated at 37°C or 50°C for 4 days. We observed developing cultures every 4 h until chains of conidia were visible (Fig. 1B and C). No differences were observed between 37°C and 50°C cultures for the first 12 h of incubation when both were making vegetative hyphae (Fig. 1B and C). Aerial hyphae emerged at 12 h in both conditions as well. Following the emergence of aerial hyphae, growth rates and morphologies showed drastic differences between 37°C and 50°C conditions. At 37°C, we observed the end of stalk elongation and vesicle formation at 16 h, presence of vesicles at 24 h, and observable conidial production by 28 h, consistent with previous reports (32). At 50°C, the end of conidiophore stalk elongation and vesicle/phialide formation for most of the population occurred at 28 h with observable conidial production by 48 h. Aerial hypha development was slower and less synchronous at 50°C than at 37°C, and 50°C conidiophores had longer, thinner stalks, smaller vesicles, and produced fewer conidia (Fig. 1B and C).

To quantify conidia produced after 4 days of incubation at 37°C or 50°C, we harvested conidia from plates in water and quantified them by hemocytometer (Fig. 1D). The 37°C plates consistently yielded more conidia compared to 50°C plates (Fig. 1D), with the 37°C producing approximately 1.3 × 10^9^ conidia/mL, and 50°C producing roughly 2 × 10^8^ conidia/mL, respectively (Fig. 1D). We also observed smaller colonies with radial growth assays at 50°C compared to 37°C in *A. fumigatus* strains CEA10 and Af293 (33, 34) (Fig. S1A). The conidial phenotype of CEA10 was analyzed by flow cytometry to determine conidial area (Fig. 1E; Fig. S1B through D). The average area of conidia produced at 50°C was larger than those produced at 37°C, with median areas of approximately 27 and 24 μm^2^, respectively (Fig. 1E; Table S1). CEA10 conidia produced at 37°C and 50°C showed significant differences in germination 5 h after exposure to carbon-containing medium, with the 50°C conidia establishing isotropic growth, germ tube emergence, and resuming nuclear division faster than the conidia produced at 37°C (Fig. S1C), consistent with results from previous studies (25, 27). We also microscopically examined conidiophores and conidia produced at 37°C and 50°C by *A. fumigatus* strains Af293 (33, 34) A1241 (AfIR964), and A1237 (AfIR97) (35) and observed elongated stalks and larger conidia at 50°C compared to 37°C in all three strains (data not shown).

### The conidial phenotype is determined during conidiophore development and is unalterable once established

**Fig 2:**
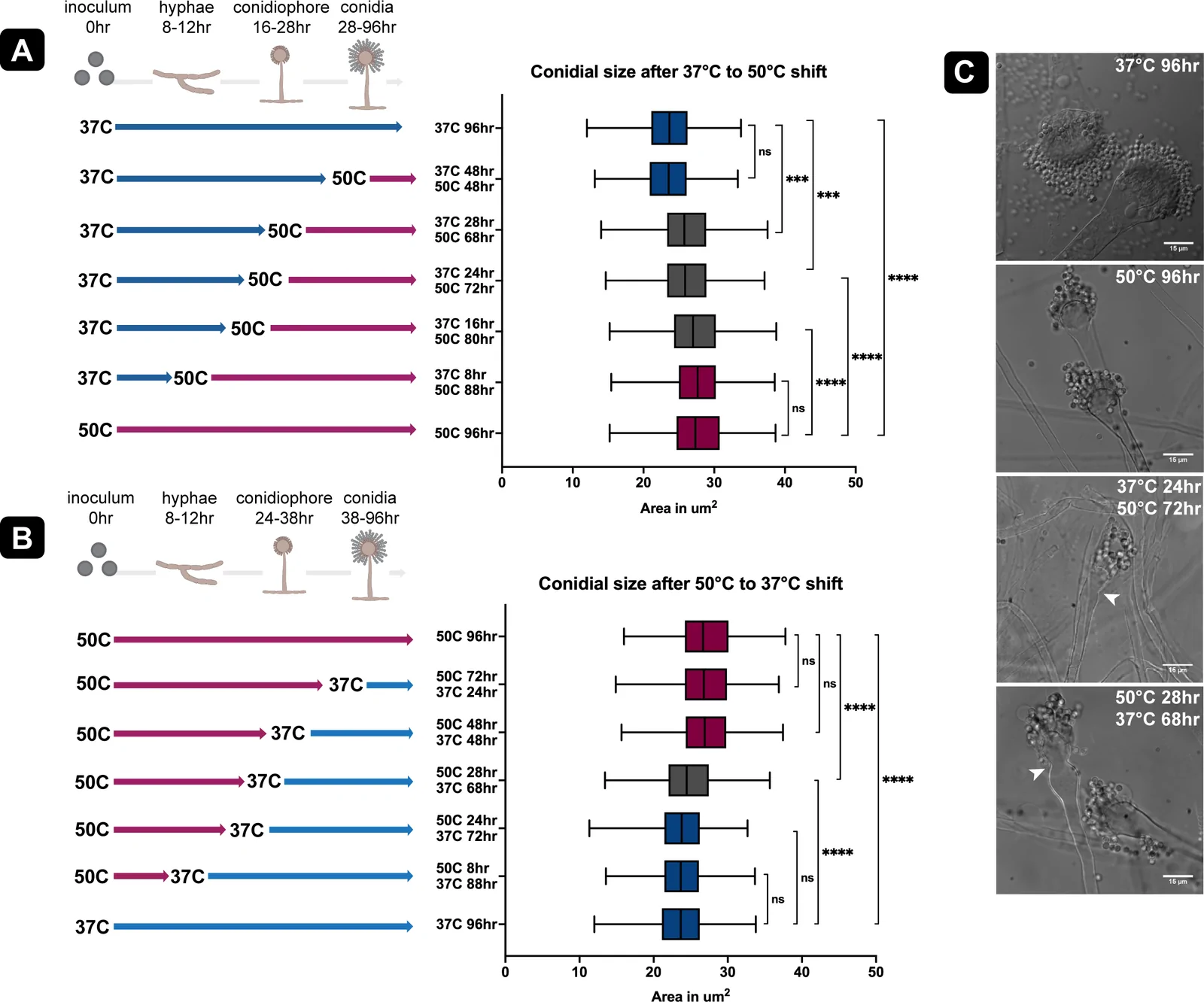
Conidial phenotype is determined during conidiophore development. (Left) Temperature swaps during specified developmental milestones: (**A**) 37–50°C transfers and (**B**) 50–37°C. (Right) Conidia were collected after 96-h incubation, and cell area was measured with flow cytometry. The *x*-axis shows area of cells in µm^2^. The middle line represents the median cell area. Statistical significance was determined by a two-tailed *t*-test (Mann-Whitney, nonparametric). *P* values are indicated by stars: ***P < 0.001, ****P < 0.0001; ns denotes not significant. Representative data of three replicates are plotted (*n* = 1,500–3,000 per sample). (**C**) Representative conidiophore morphologies after 96-h incubation. White arrow indicates abnormal vesicle development in temperature-shifted cultures.

We next investigated whether there might be sensitive phases of the asexual lifecycle in which exposure to 37°C or 50°C caused the differences in conidial size and whether there might be a commitment point after which a change in temperature would not change the conidial phenotype. To detect possible sensitive phases and commitment points, we shifted CEA10 cultures from 37°C to 50°C at defined developmental landmarks (Fig. 1B and C), incubated for 96 h, and measured the resulting conidia with flow cytometry (Fig. 2A and B). Transferring plates from 37°C to 50°C during hyphal growth (8 h) resulted in large conidia indistinguishable from conidia produced with 50°C incubation for the entire lifecycle (96 h) (Fig. 2A). Transferring plates from 37°C to 50°C early in conidiophore development (16 h) resulted in mostly large conidia with some smaller conidia also present. Transferring plates from 37°C to 50°C later in conidiophore development (24–28 h) also resulted in a mix of large and small conidia with a shift toward more small conidia. Transferring plates from 37°C to 50°C after completion of conidiophore development during conidial production (48 h) resulted in small conidia indistinguishable from those produced entirely at 37°C (96 h).

We also performed temperature shift experiments in the opposite direction, transferring cultures from 50°C to 37°C (Fig. 2B). Considering the slower development at 50°C (Fig. 1C), our results gave an almost identical pattern as the 37–50°C shift experiments (Fig. 2A). Transferring plates from 50°C to 37°C during hyphal growth (8 h) or early in conidiophore development (24 h) resulted in small conidia indistinguishable from conidia made on plates incubated entirely at 37°C (96 h). Transferring plates from 50°C to 37°C later in conidiophore development (28 h) showed a mix of large and small conidia. Transferring plates from 50°C to 37°C after conidiophore development was complete and conidia were being produced (48 and 72 h) resulted in large conidia indistinguishable from conidia from colonies incubated entirely at 50°C (96 h). Intriguingly, when the temperature was shifted during late conidiophore development, we also observed some conidiophores with abnormal vesicles and swollen phialides (Fig. 2C).

Our results suggest that environmental input during late conidiophore development (vesicle and phialide formation) determines the conidial phenotype and so defines the environmentally sensitive phase of conidial development. Our results further suggest that the conidial phenotype is not alterable once conidia begin to emerge from phialides, defining the commitment point of the conidial phenotype. These patterns hold true whether beginning at 37°C and shifting to 50°C or beginning at 50°C and shifting to 37°C (Fig. 2B). The distinct conidiophore morphologies at 37°C vs 50°C (Fig. 1B and C) and conidial morphologies that resulted from temperature shift experiments (Fig. 2C) suggest that the conidiophores themselves also have an environmentally sensitive phase either before or early in aerial hypha emergence.

### Hyphae, conidiophores, and conidia have distinct transcriptomes at 37°C vs 50°C

**Fig 3:**
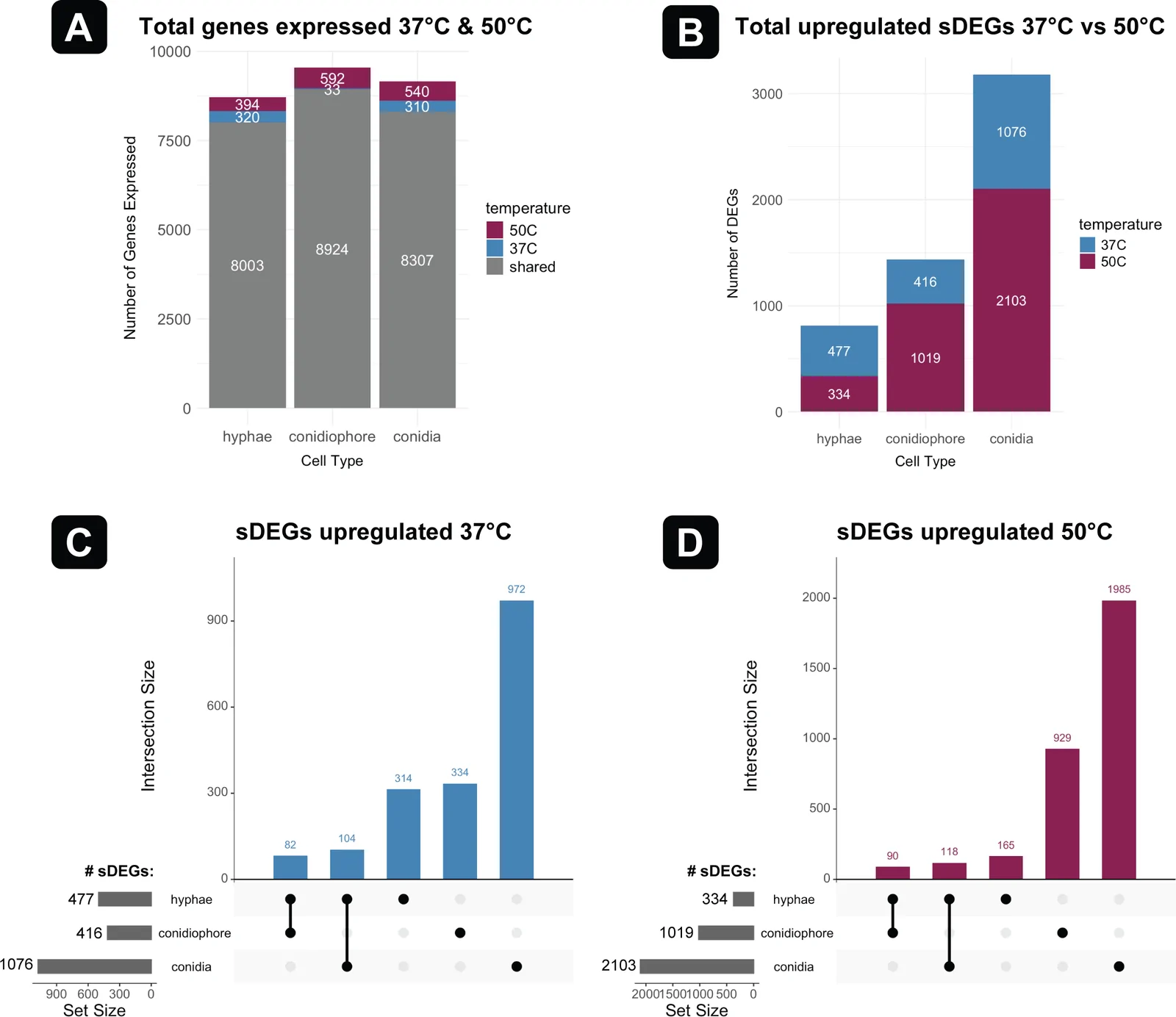
Hyphae, conidiophores, and conidia have distinct transcriptomes at 37°C vs 50°C. (**A**) Total genes expressed in hyphae, conidiophores, and conidia at 37°C and 50°C. Magenta denotes genes expressed only at 50°C. Blue denotes genes expressed only at 37°C. Gray denotes genes expressed at both temperatures. White denotes the total number of genes for each category. (**B**) Significant differentially expressed genes (sDEGs) in hyphae, conidiophores, and conidia at 37°C vs 50°C. (**C**) Upregulated sDEGs at 37°C. (**D**) Upregulated sDEGs at 50°C.

To better understand the mechanisms underlying the temperature-sensitive phase and phenotypic commitment point of conidia, we developed a method for isolating conidiophores from hyphae that used aerial growth through a thin layer of water agar followed by careful shearing with a razor (Fig. S2). We then extracted RNA from isolated hyphae, conidiophores, or conidia produced at each temperature, and analyzed the transcriptomes. We performed pairwise comparisons of total genes expressed in each cell type at 37°C or 50°C (Fig. 3A; Table S2). Of 8,717 genes expressed in hyphae, most (8,003; 91.8%) were expressed both at 37°C and 50°C, with far fewer expressed only at 37°C (320; 3.6%), or only at 50°C (394; 4.5%). Of 9,549 genes expressed in conidiophores, most (8,924; 93.5%) were expressed at both 37°C and 50°C, with far fewer expressed only at 37°C (33; 0.3%), or only at 50°C (592; 6.2%). Of 9,157 genes expressed in conidia, most (8,307; 90.7%) were expressed at both 37°C and 50°C, with far fewer expressed only at 37°C (310; 3.4%), or only at 50°C (540; 5.9%). Since the largest transcriptional differences between each cell type at both temperatures were in the conidiophores, we speculated that the conidial phenotypic program determined during late conidiophore development is transcriptionally driven.

We next identified genes with significant differentially expressed genes (sDEGs) between 37°C vs 50°C in hyphae, conidiophores, and conidia using DESeq2 with filtering based on an adjusted *P* value (*P*adj) of <0.05 and log2 fold change > 2 (Fig. 3B through D; Table S3). Our results showed that hyphae had the least sDEGs (811), followed by conidiophores (1,435), and conidia had the most sDEGs (3,179) (Fig. 3B). Based on our results showing that shifting temperature during hyphal growth does not impact asexual development (Fig. 1B, C, 2A and B), we removed all upregulated sDEGs that were shared with hyphae for 37°C and 50°C, respectively. Removal of upregulated hyphal sDEGs yielded 334 conidiophore and 972 conidial genes significantly upregulated at 37°C (Fig. 3C), and 929 conidiophore and 1,985 conidial genes significantly upregulated at 50°C (Fig. 3D; Table S3). The resulting hyphal-removed conidiophore sDEGs and conidial sDEGs were further analyzed to determine regulation of conidial production at 37°C and 50°C.

### 37°C conidiophores are upregulated in *brlA* while 50°C conidiophores are upregulated in *MAT1-1-1*

To identify genes that could be important in the environmentally sensitive phase for conidial development, we analyzed significant differentially expressed genes (sDEGs) between 37°C and 50°C conidiophores. In gene ontology (GO) enrichment analysis of upregulated sDEGs (36), the top biological processes for 37°C conidiophores included genes associated with peroxisome synthesis, transmembrane transport, and alkaloid metabolism (Fig. S3A and B). The top biological processes for 50°C conidiophores included genes associated with secondary metabolism, mycotoxin production, and gliotoxin biosynthesis (Fig. S3C and D). The enrichment for genes associated with fumagillin/pseurotin biosynthetic gene clusters (BGCs) (37, 38) in 50°C conidiophores was surprising because these secondary metabolites have been shown to occur during sexual development, though some studies have also found them in asexual development depending on conditions (20, 39).

**Fig 4:**
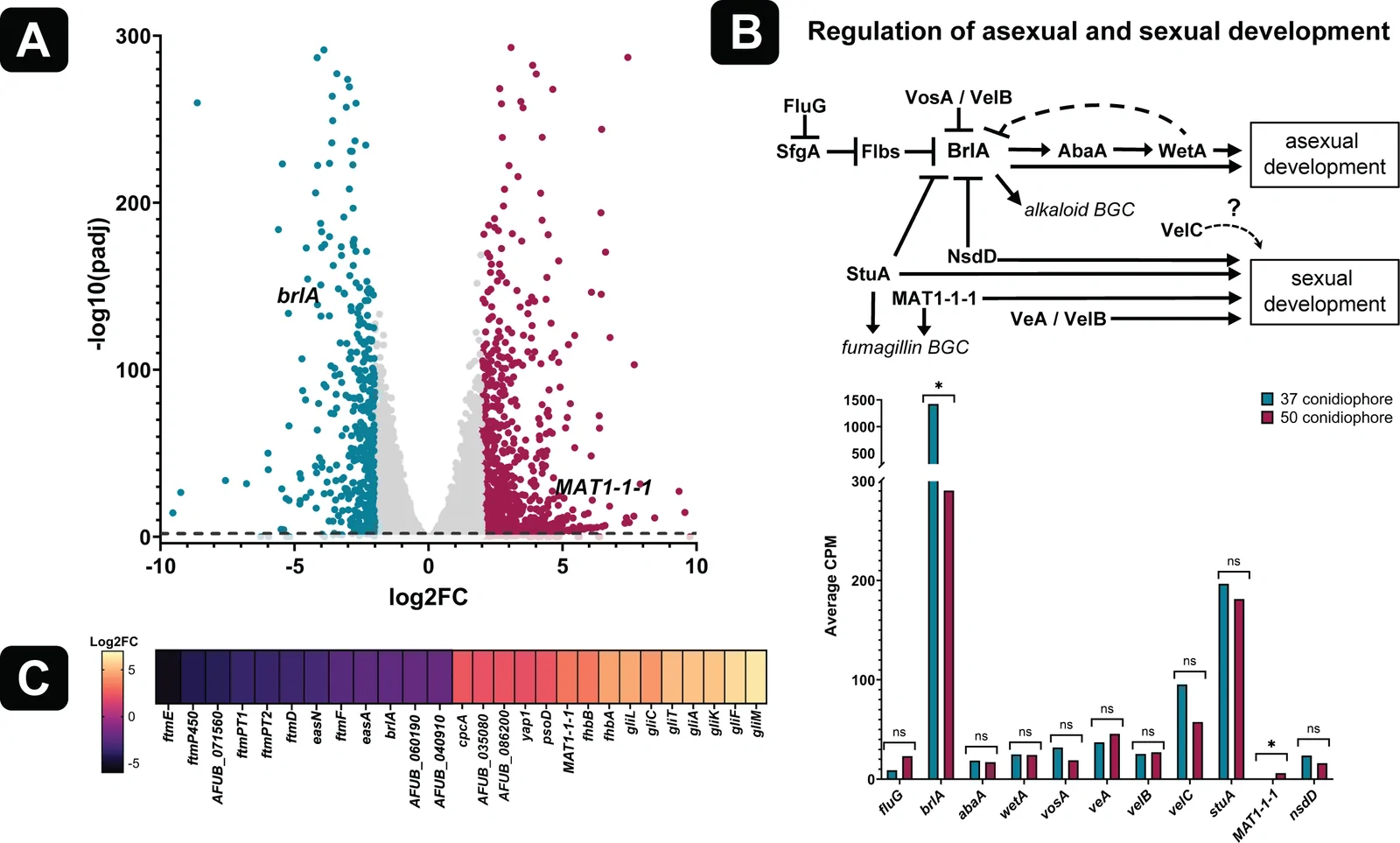
*brlA* is upregulated in 37°C conidiophores while *MAT1-1-1* is upregulated in 50°C conidiophores. (**A**) Volcano plot of 37°C vs 50°C conidiophore sDEGs with log2 fold change (Log2FC) and adjusted *P* value [−log10(*P*adj)]. Blue denotes 37°C upregulated sDEGs (416 total); magenta, 50°C upregulated sDEGs (1,019 total); gray and below dashed line, not significant (filtered |Log2FC| < 5). (**B**) Top: schematic diagram of the genetic regulation of asexual and sexual development. Bottom: transcript levels of regulators of asexual and sexual development (average CPM of three biological replicates). Blue indicates average CPM at 37°C; magenta indicates average CPM at 50°C. The star indicates significant differential expression between 37°C and 50°C conidiophores; ns indicates not significant. (**C**) Heatmap of conidiophore sDEGs according to increasing Log2FC. Negative values (purple/black) indicate upregulation in 37°C conidiophores, positive values (orange/yellow) indicate upregulation in 50°C conidiophores. Detailed descriptions of genes in the heatmap are in Table 1.

The unexpected enrichment of secondary metabolite transcripts associated with sexual development in 50°C conidiophores prompted us to examine the expression of known regulators of both asexual and sexual development in 37°C and 50°C conidiophores (Fig. 4B; Table S3). The upregulated genes in 37°C conidiophores included the master regulator of asexual development, *brlA* (AFUB_015960) (5, 6) (Fig. 4A and C; Table 1; Table S3). In contrast, other regulators of asexual development showed very similar expression levels in 37°C vs 50°C conidiophores (Fig. 4B). Members of the fumigaclavine C BGC and alkaloid BGC, known downstream targets of BrlA, were also upregulated in 37°C conidiophores (Fig. 4B and C; Table 1), including *easA* (AFUB_033650), FtmPT1 (AFUB_086330), FtmPT2 (AFUB_086290), FtmP450 (AFUB_086300), FtmE (AFUB_086320), FtmD (AFUB_086340) (9–11). The upregulation of *brlA*, fumigaclavine C BGC, and alkaloid secondary metabolite pathways in 37°C conidiophores indicated that the well-characterized asexual development pathway was active.

**TABLE 1:** 37°C vs 50°C conidiophore upregulated sDEGs^*a*^

Gene ID^*b*^	Gene	Description^*c*^	References
37°C conidiophore upregulated sDEGs			
AFUB_015960	*brlA*	Central regulator of asexual development: activator of conidiation	(5, 6)
AFUB_040910	NA^*d*^	Predicted role in asexual sporulation resulting in formation of a cellular spore, establishment of cell polarity	NA
AFUB_060190	NA	Predicted plasma membrane protein, predicted roles in regulation of conidium formation	NA
AFUB_086330	*FtmPT1*	Brevianamide F prenyltransferase FtmPT1, fumitremorgin biosynthetic process	(9–11)
AFUB_086290	*FtmPT2*	12-Alpha,13-alpha-dihydroxyfumitremorgin C prenyltransferase FtmPT2, fumitremorgin biosynthetic process	(9–11)
AFUB_086300	*FtmP450*	FtmP450 fumitremorgin C monooxygenase, fumitremorgin biosynthetic process	(9–11)
AFUB_086340	*ftmD*	O-methyltransferase FtmD, fumitremorgin biosynthetic process	(9–11)
AFUB_086320	*ftmE*	Cytochrome P450 FtmE, fumitremorgin biosynthetic process	(9–11)
AFUB_086310	*ftmF*	Verruculogen synthase FtmF, fumitremorgin biosynthetic process	(9–11)
AFUB_033650	*easA*	NADH oxidase EasA, ergot alkaloid biosynthetic process	(9–11)
AFUB_033710	*easN*	Fumigaclavine B O-acetyltransferase EasN, ergot alkaloid biosynthetic process	(9–11)
50°C conidiophore upregulated sDEGs			
AFUB_042900	*MAT1-1-1*	*A. fumigatus* mating-type protein MAT1-1-1	(18, 20)
AFUB_099650	*fhbA*	NO response FhbA, contains MAT1.1-binding motif. Regulator of sexual development	(19, 21)
AFUB_081400	*fhbB*	FhbB, regulator of sexual development	(21, 40)
AFUB_035080	NA	Predicted role in cleistothecium development, sexual sporulation resulting in formation of a cellular spore	NA
AFUB_086200	NA	Putative beta-ketoacyl synthase. Predicted roles in pseurotin A biosynthetic process	NA
AFUB_086010	*psoD*	PsoD P450 monooxygenase, fumagillin/pseurotin biosynthesis	(20, 39)
AFUB_069420	*cpcA*	CpcA, transcriptional activator of cross-pathway control system of amino acid biosynthesis	(41)
AFUB_075760	*gliA*	GliA, gliotoxin biosynthetic process	(42)
AFUB_036230	*gliC*	GliC, gliotoxin biosynthetic process	(42)
AFUB_075780	*gliF*	GliF, gliotoxin biosynthetic process	(42)
AFUB_075750	*gliK*	GliK, gliotoxin biosynthetic process	(42)
AFUB_075690	*gliL*	GliL, gliotoxin biosynthetic process	(42)
AFUB_036280	*gliM*	GliM, gliotoxin biosynthetic process	(42)
AFUB_075790	*gliT*	GliT, gliotoxin biosynthetic process	(42)
AFUB_075990	*yap1*	Yap1, regulator of oxidative stress. Important in sensing and producing gliotoxin	(42)

^*a*^ Upregulated significant differentially expressed genes (sDEGs) from 37°C vs 50°C conidiophore differential analyses that are listed in Fig. 4C heatmap.

^*b*^ Gene ID from FungiDB for *Aspergillus fumigatus* strain A1163.

^*c*^ Description simplified from FungiDB predicted protein function and literature references.

^*d*^ NA, not applicable.

To our surprise, the upregulated genes in 50°C conidiophores included *MAT1-1-1* (AFUB_042900), which encodes the master regulator of sexual development, MAT1-1-1 (Fig. 4A and C; Table 1; Table S3) (18, 21). Transcript levels of other known regulators of sexual development showed similar expression levels in 37°C vs 50°C conidiophores (Fig. 4B). Numerous targets of MAT1-1-1 containing MAT1.1 DNA-binding motifs, including members of the Fumagillin/pseurotin and gliotoxin BGCs (37, 38) and *fhbA* (AFUB_099650) and *fhbB* (AFUB_081400) (19, 21) (Fig. 4C), were upregulated in 50°C conidiophores (Fig. 4C; Table 1). Notably, *MAT1-1-1* was not differentially expressed between the 37°C vs 50°C conidia or hyphae, indicating that upregulation of *MAT1-1-1* at 50°C is limited to the conidiophore. The upregulation of *MAT1-1-1* and the downstream targets of MAT1-1-1 in 50°C conidiophores suggest that an alternative developmental pathway might be active at the higher temperature.

### 37°C conidia express higher levels of BrlA targets and dormancy regulators

To identify genes that could be important in the commitment point for conidial development, we analyzed conidial genes whose expression was significantly different between 37°C vs 50°C (sDEGs). In GO term enrichment analyses (36) of sDEGs upregulated in 37°C conidia, the top biological GO processes included carbon and acetate utilization, response to light, regulation of spore-bearing structures, and the biosynthesis of several sugars including trehalose (Fig. S4A and B), all consistent with processes known to be important in making dormant asexual spores (conidia) or early conidial germination (8, 43).

**Fig 5:**
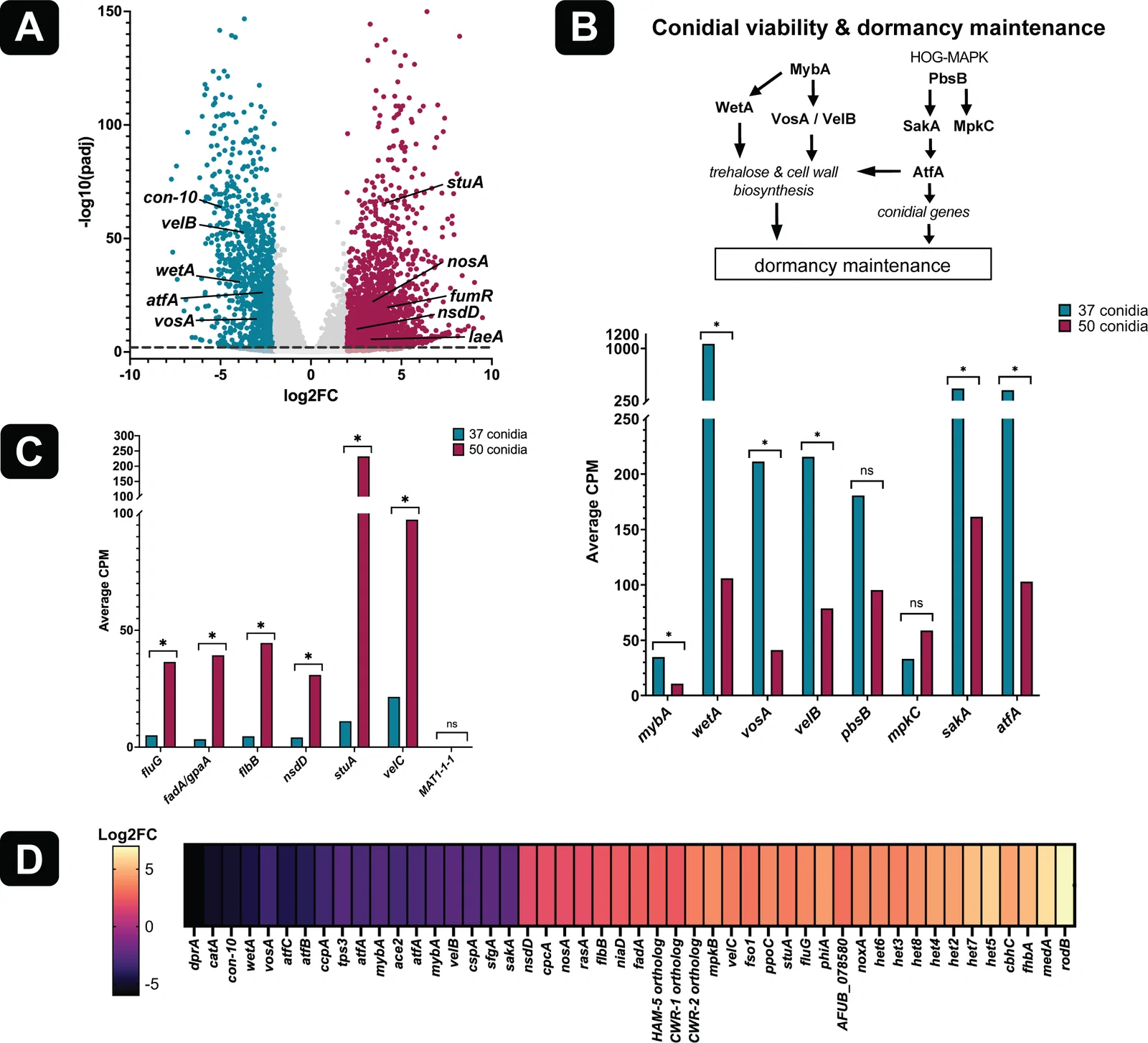
Targets of BrlA and regulators of dormancy are upregulated in 37°C conidia, while MAT1-1-1 targets and anastomosis genes are upregulated in 50°C. (**A**) Volcano plot of 37°C vs 50°C conidia sDEGs with log2 fold change (Log2FC) and adjusted *P* value [−log10(*P*adj)]. Blue denotes 37°C upregulated sDEGs (1,076 total); magenta denotes 50°C upregulated sDEGs (2,103 total); gray and below the dashed line, not significant (filtered |Log2FC| < 5). (**B**) Top: schematic diagram of the genetic regulation of viability and dormancy maintenance in conidia. Bottom: transcript levels of regulators of viability and dormancy (average CPM of three biological replicates). Blue: 37°C. Magenta: 50°C. The star indicates significant differential expression between 37°C and 50°C conidia; ns indicates not significant. (**C**) Transcript levels of negative regulators of asexual development and positive regulators of sexual development (average CPM of three biological replicates). Blue: 37°C/ Magenta: 50°C. The star indicates significant differential expression between 37°C and 50°C conidia; ns indicates not significant. (**D**) Heatmap of conidial sDEGs according to increasing Log2FC. Negative values (purple/black) indicate significant upregulation in 37°C conidia; positive values (orange/yellow) indicate significant upregulation in 50°C conidia. Detailed descriptions of genes in the heatmap are in Table 2.

Based on the upregulation of *brlA* in the 37°C conidiophores (Fig. 4A through C; Table S3), we expected that downstream targets of BrlA would be upregulated in 37°C conidia vs 50°C conidia. Indeed, numerous downstream targets of BrlA were significantly upregulated in 37°C conidia, including *wetA* (AFUB_070140) (44) and velvet regulators *vosA* (AFUB_067940) and *velB* (AFUB_002350) (5, 6, 45) (Fig. 5A and B; Table 2; Table S3). Previous studies found that WetA regulates trehalose biosynthesis and cell wall integrity, playing vital roles in conidial viability during dormancy across *Aspergillus* species (8, 46, 47). In addition to their importance during conidiation, VosA and VelB together play critical roles in conidial dormancy (45). The VosA-VelB heterodimer inhibits germination and is important in trehalose production, consistent with our GO enrichment findings (Fig. S4A and B). Other regulators of conidial viability and dormancy maintenance were upregulated in 37°C conidia (Fig. 5A through D; Table 2; Table S3), including members of the mitogen-activated protein kinase (HOG-MAPK) pathway including MAPK SakA (AFUB_012420) (47) and its downstream transcription factor AtfA (AFUB_037850). Conidial dormancy is regulated by AtfA, which inhibits germination and controls 63% of all conidial transcripts (26). Many downstream targets of AtfA were also upregulated in 37°C conidia, including *atfB* (AFUB_060680) (48), *atfC* (AFUB_016730) (49), *cspA* (AFUB_040120) (50), *drpA* (AFUB_101360) (51), *catA* (AFUB_094400) (52), and *con-10* (AFUB_095090) (Fig. 5D; Table 2; Table S3). The transcriptional driver of conidial survival MybA (AFUB_041990) (7) was also upregulated, which functions upstream of VelB-VosA and WetA that in turn upregulate trehalose biosynthesis and cell wall genes (7, 47). 37°C conidia expressed higher levels of BrlA targets and known conidial dormancy genes, consistent with 37°C conidiophores’ upregulation of *brlA*, the master regulator of asexual development.

**TABLE 2:** 37°C and 50°C conidia upregulated sDEGs involved in development^*a*^

Gene ID^*b*^	Gene	Description^*c*^	References
37°C conidia upregulated sDEGs			
AFUB_051340	*sfgA*	C6 transcription factor SgfA. Involved in regulating conidiation initiation	(53)
AFUB_070140	*wetA*	Central regulator of asexual development and spore maturation WetA. Important in conidia wall formation and trehalose biosynthesis. Regulated by BrlA	(5, 44, 45)
AFUB_012420	*sakA*	Protein kinase SakA, in HOG-MAPK pathway and CWI pathway	(26, 47)
AFUB_037850	*atfA*	Transcription factor AtfA. Regulator of dormancy maintenance	(26)
AFUB_060680	*atfB*	Transcription factor AtfB. Conidial stress response and secondary metabolism. Regulated by AtfA	(48, 49)
AFUB_016730	*atfC*	Transcription factor AtfC. Regulated by AtfA	(49)
AFUB_002350	*velB*	Asexual developmental regulator and velvet protein VelB. Required for conidial maturation	(5, 6, 45)
AFUB_067940	*vosA*	Asexual developmental regulator and velvet protein VosA	(6, 45)
AFUB_041990	*mybA*	Transcription factor MybA. Involved in conidiation, conidial cell wall biosynthesis, trehalose biosynthesis, and conidial viability	(7, 47)
AFUB_101360	*dprA*	Dehydrin protein DrpA. Involved in conidial stress response and HOG-MAPK. Regulated by AtfA	(51)
AFUB_095090	*con-10*	Conidiation-specific protein Con-10 (ConJ ortholog). Regulated by AtfA	(26, 54)
AFUB_094400	*catA*	Conidial specific catalase; important in response to reactive oxygen species and development. Regulated by AtfA	(26, 52)
AFUB_040120	*cspA*	Conidial cell wall protein CspA; important in conidial cell wall structure and trehalose biosynthesis. Regulated by MybA	(50)
AFUB_013160	*ccpA*	Conidial cell wall protein A CcpA. Important in the conidial surface structure	(55)
AFUB_089470	*tps3*	Predicted alpha, alpha-trehalose phosphate synthase subunit Tsp3. Involved in trehalose biosynthesis	(56)
AFUB_037920	*ace2*	Transcription factor Ace2. Positive regulator of asexual sporulation and spore wall assembly. Important in conidial pigmentation and virulence	(57)
50°C conidia upregulated sDEGs			
AFUB_035330	*nsdD*	Transcription factor NsdD. Positive regulator of sexual sporulation and secondary metabolism. Regulated by MAT1-1-1	(20, 21, 40)
AFUB_031050	*fluG*	Transcription factor FluG. Upstream negative regulator of asexual conidiation	(58)
AFUB_031740	*flbB*	Transcription factor FlbB. Upstream negatively regulator of asexual conidiation	(58)
AFUB_012620	*fadA*	Guanine nucleotide binding protein (G-protein), alpha subunit FadA (GpgA homolog). Positive regulator of vegetative growth, regulated by MAT1-1-1	(21)
AFUB_099650	*fhbA*	Nitric oxide (NO) response FhbA. Contains MAT1.1-binding motif. Regulator of sexual reproduction	(21)
AFUB_066820	*nosA*	Transcription factor NosA. Roles in cleistothecium development and ascospore formation	(59)
AFUB_016640	*rodB*	Conidial hydrophobin, RodB. Contains MAT1.1-binding motif	(21, 32)
AFUB_023920	*stuA*	APSES-domain containing transcription factor StuA. Hyphal competency switch, repressor of *brlA* during conidiation initiation. Positive regulator of sexual development	(32)
AFUB_028890	*medA*	Transcription factor MedA. Important in phialide formation during conidiation	(60)
AFUB_012300	*niaD*	Nitrate reductase NiaD. Regulated by FhbA	(21, 40)
AFUB_001420	*noxA*	NADPH oxidase NoxA. Positive regulator of cleistothecium development	(61)
AFUB_066880	*velC*	Velvet regulator VelC. *Aspergillus nidulans* VelC ortholog positively regulates sexual development	(32, 62)
AFUB_045170	*phiA*	Cell wall protein PhiA. Role in asexual and sexual sporulation, regulated by StuA	(32)
AFUB_078580	NA	Predicted roles in cleistothecium development	NA
AFUB_017010	*het2*	Heterokaryon incompatibility	(63)
AFUB_028340	*het3*	Heterokaryon incompatibility	(63)
AFUB_046770	*het4*	Heterokaryon incompatibility	(63)
AFUB_045110	*het5*	Heterokaryon incompatibility	(63)
AFUB_078080	*het6*	Heterokaryon incompatibility	(63)
AFUB_085570	*het7*	Heterokaryon incompatibility	(63)
AFUB_080400	*het8*	Heterokaryon incompatibility	(63)
AFUB_080180	NA	Predicted heterokaryon incompatibility	NA
AFUB_051520	NA	*N. crassa* CWR-1 (NCU01380) ortholog. Predicted protein of unknown function	(64)
AFUB_030130	NA	*N. crassa* CWR-2 (NCU01382) ortholog. Predicted protein of unknown function	(64)
AFUB_078810	*mpkB*	Mitogen-activated protein (MAP) kinase MpkB. *N. crassa* MAK-2 (NCU02393) ortholog	(65)
AFUB_046510	*cbhC*	Cellulose binding protein CbhC. *N. crassa* NCU07190 *doc* locus (self-recognition) ortholog	(65)
AFUB_072440	*fso1*	Fso1, protein of unknown function. *N. crassa* SOFT (NCU02794) ortholog	(65)
AFUB_061660	NA	*N. crassa* HAM-5 (NCU01789) ortholog. Predicted protein of unknown function	(65)
AFUB_058790	*rasA*	RAS signaling pathway GTPase RasA; regulates development and cell wall integrity	(66, 67)

^*a*^ Upregulated significant differentially expressed genes (sDEGs) from 37°C vs 50°C conidial differential analyses listed in Fig. 5D heatmap.

^*b*^ Gene ID from FungiDB for *Aspergillus fumigatus* strain A1163.

^*c*^ Description simplified from FungiDB predicted protein function and literature references.

### 50°C conidia express higher levels of MAT1-1-1 targets, sexual developmental regulators, and anastomosis homologs

We reasoned that the 50°C conidial sDEGs would uncover clues on dormancy maintenance in 50°C conidia. The top biological GO processes (36) of sDEGs upregulated in 50°C conidia vs 37°C included cytoskeleton synthesis and organization, cell cycle, nuclear processes, and fumagillin biosynthesis (Fig. S4C and D), processes in which take part in the formation of sexual spores (ascospores) within the asci in sexual reproductive structures (cleistothecia) or have been associated with sexual reproduction (20, 39, 63). Previous work found that MAT1-1-1 regulates fumagillin BGCs (20), and that fumagillin metabolites are produced during both sexual development and cytoskeletal remodeling (68). Several genes including members of the fumagillin biosynthesis and BGC (37, 38) and were upregulated in 50°C conidia, including the positive regulator of fumagillin biosynthesis FumR (AFUB_086150) (69, 70) (Fig. 5A; Table 3; Table S3). Additionally, chromatin remodeler and global regulator of secondary metabolism LaeA (AFUB_014200) (71) was upregulated, suggesting that LaeA may play an important role in the production of fumagillin and gliotoxin associated transcripts observed in the upregulated 50°C conidial sDEGs (28, 29).

**TABLE 3:** 50°C conidia upregulated sDEGs important in secondary metabolism and cell cycle^*a*^

Gene ID^*b*^	Gene	Description^*c*^	References
Secondary metabolism			
AFUB_014200	*laeA*	Chromatin remodeler LaeA. Global regulator of secondary metabolism	(71)
AFUB_046990	NA^*d*^	Beta-ketoacyl synthase. Fumagillin biosynthetic process	(39)
AFUB_086050	NA	UbiA prenyltransferase. Fumagillin biosynthetic process	(39)
AFUB_018370	NA	Beta-ketoacyl synthase. Fumagillin biosynthetic process	(39, 72)
AFUB_086150	*fumR*	Transcription factor FumR. Positive regulation of fumagillin biosynthetic process, pseurotin A biosynthetic process	(69, 70)
AFUB_086200	NA	Beta-ketoacyl synthase. Fumagillin biosynthetic process	(39, 69, 72)
AFUB_086190	*Fma-AT*	Serine aminopeptidase Fma-AT. Fumagillin biosynthetic process	(72)
AFUB_086030	*psoA*	Pseurotin A, non-ribosomal peptide synthetase (NRPS) PsoA. Fumagillin biosynthetic process	(73)
AFUB_039500	*oefC*	DNA-binding RNA polymerase II transcription factor OefC. Gliotoxin biosynthetic process	(42)
AFUB_069960	*rglT*	Transcription factor RglT. Gliotoxin biosynthetic process	(42)
AFUB_075700	*gliJ*	GliJ. Gliotoxin biosynthetic process	(42)
AFUB_075690	*gliL*	GliL. Gliotoxin biosynthetic process	(42)
AFUB_036280	*gliM*	GliM. Gliotoxin biosynthetic process	(42)
AFUB_075790	*gliT*	GliT. Gliotoxin biosynthetic process	(42)
AFUB_036300	*gliZ*	Transcription factor GliZ. Gliotoxin biosynthetic process	(42)
Cell cycle genes			
AFUB_028770	*imeB*	Meiosis induction protein kinase ImeB	(21)
AFUB_083650	*nimX*	Mitotic cyclin-dependent kinase NimX. Cdc28 ortholog	(74)
AFUB_069140	*nimE*	G2/M-specific cyclin NimE. Clb2 ortholog	(74)
AFUB_075160	*nimO*	G1/S-specific cyclin NimO	(75)
AFUB_087970	*bimC*	Kinesin BimC, required for progression through M phase	(74)
AFUB_034890	*mad2*	Mitotic spindle checkpoint protein Mad2	(74)
AFUB_009830	*nusA*	DNA repair protein NusA. Rad51 ortholog	(74)
AFUB_074100	*sldA*	SAC protein kinase and cell cycle checkpoint protein SldA. Bub1 ortholog. Prevents progression into anaphase	(74)
AFUB_090950	*aspB*	Septin protein AspB. Cdc3 ortholog	(74)
AFUB_056080	*aspA*	Septin protein AspA. Cdc11 ortholog	(74)
AFUB_053640	*swi5*	DNA repair protein Swi5	(74)
AFUB_056360	*hexA*	Woronin body protein HexA	(76)
AFUB_010330	*ftsZ*	Beta tubulin, FtsZ. Microtubule depolymerization, mitotic spindle disassembly	NA
AFUB_098620	NA	Predicted regulation in meiosis and DNA replication	NA
AFUB_028000	*spo22*	Meiosis specific protein Spo22. ZIP4 and TEX11 ortholog	NA
AFUB_015890	NA	Centromere protein X. Predicted roles in kinetochore assembly and DNA repair	NA
AFUB_030640	NA	Predicted roles in meiosis and ascospore formation	NA

^*a*^ Genes involved in secondary metabolism and cell cycle that are 50°C upregulated significant differentially expressed genes (sDEGs) from 37°C vs 50°C conidial differential analyses.

^*b*^ Gene ID from FungiDB for *Aspergillus fumigatus* strain A1163.

^*c*^ Description simplified from FungiDB predicted protein function and literature references.

^*d*^ Not applicable.

Though MAT1-1-1 is typically involved in sexual development rather than asexual development, the upregulation of *MAT1-1-1* in 50°C conidiophores (Fig. 4A through C; Table 1; Table S3) suggested that downstream targets of MAT1-1-1 might be upregulated in 50°C conidia. Indeed, many downstream targets of MAT1-1-1 were differentially upregulated in 50°C conidia vs 37°C conidia, including numerous genes important in sexual reproduction and cell cycle genes (Fig. 5A through D; Tables 2 and 3). Several genes upregulated in 50°C conidia contain MAT1.1-binding motifs and are associated with sexual development, including *nsdD* (AFUB_035330) (21), *fhbA* (AFUB_099650) (40), *rodB* (AFUB_016640) (17), and *fadA* (AFUB_012620) (21) (Fig. 5C and D; Table 2). Although not directly driven by MAT1-1-1, transcript levels of numerous positive regulators of cleistothecium formation were also upregulated in 50°C conidia, including transcription factor StuA (AFUB_023920) (32, 77) transcription factor NosA (AFUB_066820) (59), and NADPH oxidase NoxA (AFUB_001420) (61). MAT1-1-1 has also been reported to regulate cell cycle genes (20), and the 50°C conidial sDEGs included numerous regulators of cell cycle progression, including mitotic cyclin-dependent kinase NimX (AFUB_083650), G2/M-specific cyclin NimE (AFUB_069140), and G1/S-specific cyclin NimO (AFUB_075160) (74, 75) (Table 3; Table S3). Based on work in *A. nidulans*, it is assumed that dormant conidia in *A. fumigatus* are arrested in the G1 phase of the cell cycle (78); however, the abundant upregulation of cell cycle genes suggests nuclei in 50°C conidia are not fully arrested in G1. Consistent with the idea that nuclei in 37°C vs 50°C are in different states, ImageStream flow cytometry analyses showed a nuclear fluorescent signal that was less intense in 50°C conidia, indicating that nuclei are less compact compared to nuclei of 37°C conidia (Fig. S1C and D; Table 3). Strikingly, transcripts of the meiosis induction protein kinase ImeB (AFUB_028770) (21) and meiotic regulator Spo22 (AFUB_028000) were also upregulated, suggesting that meiotic processes are stimulated in conidia produced at 50°C (Table 3 and Table S3). The abundance of MAT1-1-1 targets, including known sexual development genes and cell cycle genes, is consistent with 50°C conidiophores’ upregulation of *MAT1-1-1*, the master regulator of sexual development.

In addition to sexual development genes, many regulators of anastomosis (hyphal fusion) were also upregulated in 50°C conidia vs 37°C conidia (Fig. 5D; Table 2; Table S3). In fungi, cell wall remodeling and hyphal fusion are tightly linked with self/nonself recognition; however, the underlying cell-cell communication is not well defined in *A. fumigatus*. In *Neurospora crassa*, MAK-2 (NCU02393), SOFT (NCU02794), and HAM-5 (NCU01789) govern cell-cell communication by oscillating at hyphal tips before fusion (65, 79). To our surprise, the *A. fumigatus* orthologs *mpkB* (AFUB_078810), AFUB_072440, and AFUB_061660, respectively, were upregulated in 50°C conidia (Fig. 5D; Table 2; Table S3). *N. crassa* cell wall remodeling proteins CWR-1 (NCU01380) and CWR-2 (NCU01382) serve as checkpoints during cellular fusion (64). *A. fumigatus* CWR-1 orthologs AFUB_051520 and AFUB_030130 were also upregulated. We also found that seven previously identified heterokaryon incompatibility (*het*) genes were upregulated in 50°C conidia (Fig. 5D; Table 2; Table S3). *het* genes play roles in determining post-fusion compatibility by self-recognition (15, 16). Although anastomosis is a prerequisite for sexual reproduction, these genes are not known to be regulated by mating-type loci and appear to independently drive vegetative compatibility (20, 63). The abundance of anastomosis homologs and heterokaryon compatibility genes in the 50°C conidial sDEGs suggests that 50°C conidia may be transcriptionally predisposed for cellular fusion during germination. Furthermore, these results suggest that the difference in conidial size and dormancy breaking in conidia produced at 37°C vs 50°C are transcriptionally driven by distinct developmental pathways.

## DISCUSSION

**Fig 6:**
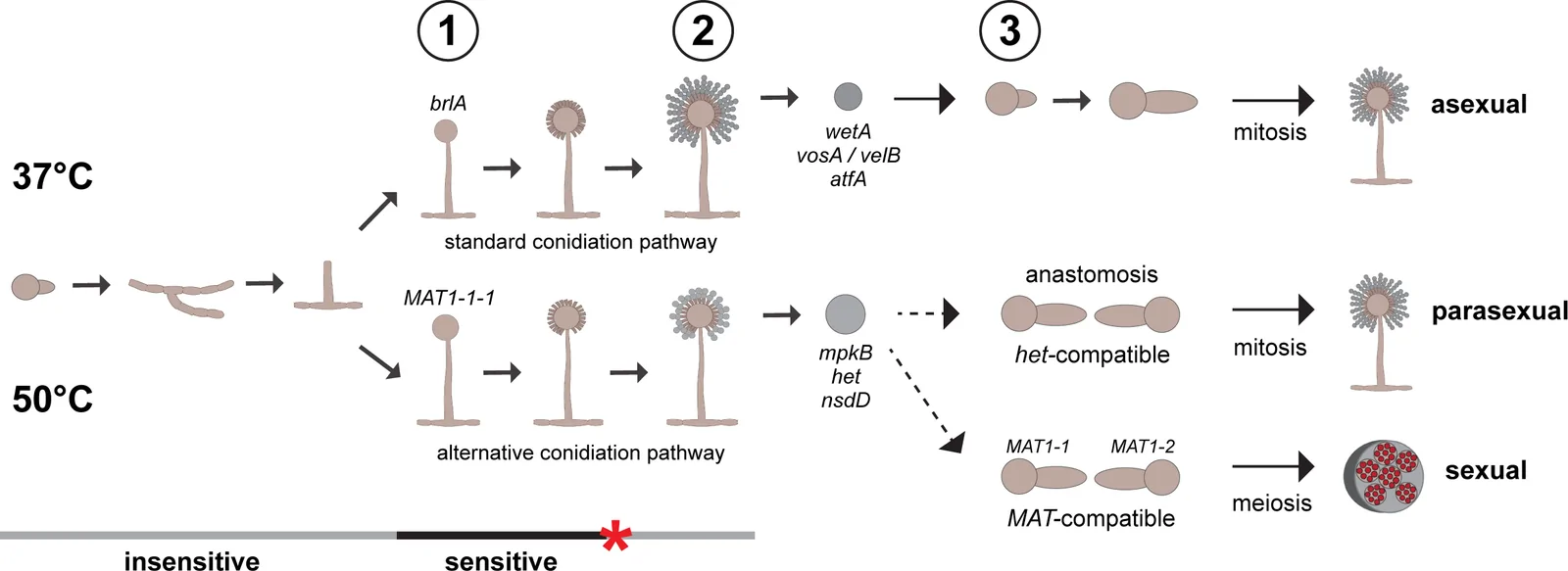
Temperature during late-stage conidiophore development stimulates distinct conidiation pathways to produce conidia that are transcriptionally primed for asexual, parasexual, or sexual reproduction (model). Comparison of development at 37°C (top) or 50°C (bottom). At both temperatures, germlings give rise to hyphae, which give rise to foot cells to start conidiation (see Fig. 1A). At both temperatures, the phenotype and transcriptome are not sensitive to temperature before or after late-conidiophore development, as indicated by gray (insensitive) and black (sensitive) lines below the diagram. (1) Late-stage conidiophores sense environmental temperature and upregulate distinct conidiation pathways (37°C *brlA*; 50°C *MAT1-1-1*). (2) After the conidial phenotypic commitment point (indicated by red asterisk), distinct conidiation pathways transcriptionally prime conidia with specific dormancy maintenance and/or germination programs. Resulting 37°C conidia are upregulated in downstream targets of BrlA (*wetA*, *vosA*, *velB*, *atfA*); 50°C conidia are upregulated in downstream targets of MAT1-1-1 (*mpkB*, *het*, *nsdD*). (3) Transcriptionally primed conidia germinate and follow either asexual (37°C), parasexual (50°C), or sexual (50°C) reproductive strategies, leading to progeny that are more likely to survive in the environment in which the parental conidiophore was formed. The dotted line indicates hypothetical reproductive outcomes upon germination. Circled numbers indicate main steps of the model. See Discussion for more details.

Previous work showed that *A. fumigatus* conidia produced at 37°C were smaller and broke dormancy more slowly than those produced at 50°C (25), and that these differences correlated with transcriptional changes (27). In this work, we investigated key landmarks in the asexual development pathway that leads to the production of conidia at 37°C and 50°C. Our temperature shift experiments showed that the conidial phenotype influenced by environmental temperature is determined during a narrow developmental window in late-stage conidiophore development (Fig. 2A through C). Our RNA-seq analyzes showed that incubation at 37°C during the late-conidiophore developmental window leads to increased expression of *brlA*, encoding the master regulator of asexual development, in conidiophores and of BrlA’s downstream targets in conidia in agreement with previous studies of asexual development (9–11). In contrast, incubation at 50°C during the late-conidiophore developmental window leads to increased expression of *MAT1-1-1*, encoding the master regulator of sexual development, in conidiophores and of MAT1-1-1’s downstream targets in conidia, including genes encoding fumagillin/pseurotin BGCs (20, 39) as well as sexual regulators and anastomosis genes. It is important to note, however, that the MAT1-1-1-dependent pheromone-encoding genes *ppgA* and *preA* (80) were not significantly differentially expressed, which may imply that the conidial phenotype resulting from conidiation at 50°C may not be solely influenced by MAT1-1-1 gene regulation. Our finding of a switch that appears to toggle between asexual, parasexual, and/or sexual lifecycles in response to temperature has important implications for the diversity and fitness of *A. fumigatus* in natural and agricultural environments. Based on our results, we propose a model in which *A. fumigatus* activates radically different developmental pathways during conidiation at 37°C vs 50°C (Fig. 6).

### Step 1: late-stage conidiophores sense environmental temperature and stimulate distinct conidiation pathways that direct conidial transcriptional programs and phenotypes

Our results suggest that late-stage conidiophore development is the environmentally sensitive developmental window and the most critical time in the asexual lifecycle for determining the phenotype of future conidia (Fig. 2A and B). Importantly, our results indicate that the conidial phenotype is not alterable once it has been established, suggesting that there is a conidial phenotypic commitment point after vesicles are formed and before conidia emerge. We propose that maturing conidiophore vesicles sense the external environment and activate specific developmental programs that ultimately direct conidial production (Fig. 6, step 1). This pattern holds true whether beginning at 37°C and shifting to 50°C (Fig. 2A) or beginning at 50°C and shifting to 37°C (Fig. 2B).

Our 37°C vs 50°C differential RNA-seq analyses showed that 37°C conidiophores are upregulated in *brlA*, which encodes the master regulator of asexual development, while 50°C conidiophores are upregulated in *MAT1-1-1*, which encodes the master regulator of sexual reproduction (Fig. 4A through C). We propose that late-stage conidiophores, which sense the 37°C environment at the conidial phenotypic commitment point, stimulate *brlA* transcription, allowing BrlA to drive the paradigmatic asexual conidiation pathway. Alternatively, conidiophores that sense the 50°C environment at the conidial phenotypic commitment point stimulate *MAT1-1-1* transcription, allowing MAT1-1-1 to drive an alternative conidiation pathway (Fig. 6, step 1). We speculate that environmental input at late-stage conidiophore development across a range of temperatures and conditions will induce regulators that direct other currently undefined alternative conidiation pathways.

### Step 2: distinct conidiation pathways transcriptionally prime conidia with specific dormancy maintenance and/or germination programs

Conidia produced at 37°C showed upregulation of transcripts encoding downstream targets of BrlA, including regulators of conidial viability and dormancy WetA, MybA (7), VelB, VosA, and AtfA (26) (Fig. 5B). Conversely, conidia produced at 50°C displayed upregulation of downstream targets of MAT1-1-1, including fumagillin/pseurotin BGCs, sexual reproduction genes, and cell cycle genes. Dormant conidia are presumed to be arrested in the G1 phase of the cell cycle (78). The striking abundance of cell cycle genes upregulated in 50°C conidia (Fig. 5B) suggests that the cell cycle might be more active compared to 37°C conidia. Indeed, the nuclei in 50°C conidia appeared to be more diffuse and completed mitosis more rapidly than 37°C conidia (Fig. S1C), consistent with these nuclei having exited a G1 arrest. This difference in cell cycle could be in preparation for meiosis, as the meiosis induction protein kinase ImeB (21) was upregulated in 50°C conidia. In addition to upregulation of sexual regulators and cell cycle genes, our differential expression results showed that 50°C conidia were upregulated in anastomosis orthologs of *N. crassa* anastomosis genes including MAK-2 (65) (*A. fumigatus* MpkB), SOFT (79), HAM-5, CWR-1, CWR-2 (20, 63, 64) as well as seven putative heterokaryon incompatibility (*het*) genes (Fig. 5D; Table 2; Table S3) (15, 16). We propose that conidia produced from 37°C or 50°C conidiophores are transcriptionally primed to maintain dormancy and/or germinate optimally at 37°C or 50°C, respectively (Fig. 6, step 2). We speculate that exposure to other environmental conditions during late-stage conidiophore development stimulates other distinct developmental pathways to produce unique conidial programs (Fig. 6, step 2) that optimize survival of progeny for their future in that environment.

### Step 3: transcriptionally primed conidia germinate and are more or less susceptible to fusion with compatible partners, favoring reproductive strategies that are optimal for survival of progeny in the environment in which they were primed

Previous work showed that conidia produced at 37°C germinate optimally at 37°C, while conidia produced at 50°C germinate faster in any condition tested (25). The upregulation in anastomosis, heterokaryon incompatibility, and sexual reproduction genes in 50°C conidia could stimulate rapid initiation of cellular fusion with compatible partners (Fig. 6, step 3). We propose that conidia produced from 37°C conidiophores are primed for asexual development, and that conidia produced from 50°C conidiophores are primed for sexual or possibly parasexual development (Fig. 6, step 3). Conidia produced from 50°C conidiophores germinate optimally at 50°C, enabling faster growth through the surrounding substrate where compatible fusion or mating partners could reside. Ascospores, the products of sexual reproduction, require a heat shock of 60–80°C for efficient germination, while conidia are killed at such high temperatures (24). Stimulating specific conidiation pathways that drive favorable reproductive programs would enhance survival of progeny in the environment in which they were primed.

### Concluding thoughts

*A. fumigatus* is thermotolerant and commonly isolated from compost heaps where temperatures routinely reach 50°C or higher (81). As others have noted, the environmental conditions in compost are similar to conditions that favor *A. fumigatus* sexual reproduction, and the temperature gradient in compost piles (20–70°C) includes internal regions that are hot enough to furnish the required heat shock for germination of any ascospores produced (13, 14, 22–24). Azole-resistant *A. fumigatus* has been found frequently in agricultural environments, including in soil, compost, and plant debris (82), and recent work has uncovered extremely high levels of meiotic recombination in *A. fumigatus* thought to have assisted in the generation and spread of alleles causing resistance to azole antifungals (63). Our results strongly support the idea previously proposed by Snelders and colleagues that compost might be the environmental niche for *A. fumigatus* sexual reproduction (22). Because a temperature above 50°C kills conidia and most competing microbes, but not ascospores, sexual development at 50°C would give *A. fumigatus* a major survival advantage in compost, as well as the potential for better adaptation of progeny because of the genetic diversity of ascospores.

Our results strongly suggest that the temperature-induced programs determined during late-stage conidiophore development are transcriptionally regulated; in addition, our results suggest that post-transcriptional regulation might also be important. Other sDEGs upregulated in 50°C conidiophores included an abundance of both non-coding RNAs (ncRNAs) and ribosomal biogenesis genes, including the 18S rRNA subunit (AFUB_090410), translation initiation factors IF6 (AFUB_053550), eIF-1A (AFUB_059560), and ribosome biogenesis proteins NOP2/RsmB (AFUB_050290) and Slx9 (AFUB_002540) (Table S3). Although ribosome biogenesis utilizes approximately 80% of cellular energy (83), increased ribosomal production drives cellular growth, stress response, and enhances environmental adaptation (84), consistent with altering growth regulation. Importantly, previous studies demonstrated that BrlA suppresses ribosome biogenesis, which is presumed to be useful in metabolic preservation during conidial production (12). Additionally, 50°C conidiophores showed upregulation of *cpcA* (AFUB_069420) (Fig. 4C; Table 1; Table S3), whose protein product drives the cross-pathway control system (41), which shifts metabolic activities as well as amino acid production in response to environmental stress. These results suggest that post-transcriptional processes might also be used during conidiophore development in response to environmental temperature to regulate distinct developmental pathways.

Although our results strongly suggest that conidia produced at 37°C or 50°C are primed for asexual or sexual development, respectively, we have only performed these experiments with the *MAT1-1* strain CEA10 (A1163). Future work will include testing anastomosis and/or mating in pairs of strains of opposite mating types. Future work will also test the range of environmental temperatures and conditions that impact conidial development and the importance of non-transcriptional regulation in this process.

## MATERIALS AND METHODS

### Strains and conidial preparation

The *Aspergillus fumigatus* wild-type strain CEA10 (A1163) was utilized for most analyses (30, 31). In addition to CEA10, Af293 (33, 34) was utilized for radial growth analyses and strains Af293 (33, 34) A1241 (AfIR964), and A1237 (AfIR97) (35) were examined microscopically for developmental phenotypes at 37°C or 50°C. Except for radial growth assays, conidia were inoculated by spreading evenly to cover standard 100 mm × 100 mm petri plates containing solid Aspergillus minimal medium (AMM) (85), incubated for 3 or 4 days at 37°C or 50°C, and harvested in 10 mL of DEPC-treated water (Invitrogen) per plate. Isolated conidia were filtered 4× through 22–25 µm Miracloth (Sigma) and washed by centrifuging for 1 min at 7,000 × *g* at 4°C and resuspended in 1 mL DEPC-treated water. Isolated conidia were quantified via hemocytometer prior to all downstream analyses. Conidia were diluted to a common concentration of 1 × 10^8^ conidia/mL for all experiments.

### Radial growth analyses

Colony radial growth was observed by point inoculating 1 µL of 1 × 10^8^ conidia/mL onto the middle of petri dishes containing solid AMM and incubating at 37°C or 50°C for 3 days. Colony diameter was measured every 24 h. Three biological replicates were performed for each condition. Data were plotted and analyzed using GraphPad Prism software version 10.

### Defining developmental milestones

Conidia were inoculated onto pre-warmed solid AMM petri plates at a final concentration of 1 × 10^7^ conidia/plate and incubated at 37°C or 50°C. Growth was monitored every 4 h via standard light microscopy. Wet mounts were analyzed for hyphal cells, and aerial conidiophore development was analyzed by slicing and removing a 0.5 × 2 cm rectangle cube from the petri plates, placing them onto microscope slides, and analyzing the side view of the aerial conidiophore growth at various time points. Three biological replicates were carried out with a minimum of 10 conidiophores observed per replicate. All three replicates gave very similar patterns.

### Temperature shift experiments

Conidia were inoculated onto pre-warmed solid AMM petri plates at a final concentration of 1 × 10^7^ conidia/plate and incubated at 37°C or 50°C. Growing cultures were transferred at defined milestones from 37°C to 50°C, or vice versa, and resulting conidia produced after 96 h were isolated, and size was analyzed using flow cytometry. Three biological replicates were carried out per experiment.

### Isolation of hyphae

Two hundred microliters of 1 × 10^8^ conidia/mL *Aspergillus fumigatus* CEA10 conidia was inoculated into 500 mL Erlenmeyer flasks containing 200 mL liquid AMM. Cultures were incubated for 12 h at either 37°C or 50°C with shaking at approximately 150 rpm. Hyphae were filtered through 22–25 µm Miracloth, washed with DEPC-treated water, and RNA extraction was immediately carried out.

### Isolation of conidiophores

Two hundred microliters of 1 × 10^8^ conidia/mL *Aspergillus fumigatus* CEA10 conidia was inoculated into 40 mL melted AMM (cooled to 50°C) and poured into 60 mm × 15 mm Petri dishes (ThermoFisher Scientific). The conidia/media mixture was allowed to harden at room temperature for 15 min, and then 4 mL of melted H_2_O agar was layered directly on top to physically isolate developing conidiophores from the underlying hyphae. After 28 and 38 h of incubation at 37°C or 50°C, respectively, the conidiophores were isolated by gently shaving off the top conidiophore layer using a razor blade. Resulting isolated conidiophores were gently scraped from the plate and viewed microscopically to ensure purity. The resulting conidiophore shavings were transferred into a 15 mL Falcon tube for RNA extractions.

### RNA extractions

Isolated cells were immediately lysed by resuspending cells in 100 µL 1× TE buffer (Invitrogen) in a 15 mL Falcon tube. Four 2.33 mm beads (BioSpec Products) and 0.1 mL (100 mg) of 425–600 µM beads (Sigma) were added and vortexed on the highest setting for 2 min. RNA extractions were carried out utilizing TRIzol according to the manufacturer’s protocol (Invitrogen).

### RNA sequencing

RNA sequencing was performed by Plasmidsaurus on RNA extracted from isolated conidia, hyphae, and conidiophores produced at 37°C or 50°C (Fig. S2). Three biological replicates of each condition were sequenced. Quality of the fastq files was assessed using FastQC v0.12.1. Reads were then quality filtered using fastp v0.24.0 with poly-X tail trimming, 3′ quality-based tail trimming, a minimum Phred quality score of 15, and a minimum length requirement of 50 bp. Quality-filtered reads were aligned to the *Aspergillus fumigatus* CEA10 (A1163) (30, 31) reference genome using STAR aligner v2.7.11 with non-canonical splice junction removal and output of unmapped reads, followed by coordinate sorting using samtools v1.22.1. PCR and optical duplicates were removed using UMI-based deduplication with UMIcollapse v1.1.0. Alignment quality metrics, strand specificity, and read distribution across genomic features were assessed using RSeQC v5.0.4 and Qualimap v2.3, with results aggregated into a comprehensive quality control report using MultiQC v1.32. Gene-level expression quantification was performed using featureCounts (subread package v2.1.1) with strand-specific counting, multi-mapping read fractional assignment, exons, and 3′ UTR as the feature identifiers, and grouped by gene_id. Final gene counts were annotated with gene biotype and other metadata extracted from the reference GTF file. Sample-sample correlations for sample-sample heatmap and PCA were calculated on normalized counts (trimmed mean of *M* values [TMM]) using Pearson correlation. Differential analyses of RNA-seq data were carried out using DESeq2 v.1.50.2 (BiocManager v1.30.27) in R (86). Low-read counts were filtered out (<5). All parameters were performed according to standard methods. Resulting differentially expressed genes (DEGs) were filtered based upon adjusted *P* value (*P*adj < 0.05) and |log2 fold change| > 2.

### Gene ontology analyses

Gene ontology (GO) enrichment analyses were performed using FungiDB (36). Fisher’s exact tests were utilized for all GO analyses (*P* value cutoff = 0.05), ranked from highest to lowest *P* value. All graphs and plots were generated using RStudio version 2025.05.0+496 and Prism version 10 (GraphPad Software). BiocManager v1.30.27, clusterProfiler v4.18.4, dplyr v.1.1.4, ggplot2 v.4.0.1, and tidyr v.1.3.2 were utilized for top 10 GO enrichment plots.

### RNA-seq data interpretation

All graphs and plots for RNA-seq and gene ontology were generated using RStudio version 2025.05.0+496 and Prism version 10 (GraphPad Software). UpSet plots were created using ComplexHeatmap v2.26.0 (BiocManager v1.30.27) and UpSetR v1.4.0. For UpSet plot construction, the resulting normalized counts from the differential analyses after filtering out low reads were used. To get the total numbers of genes expressed by each cell type (hyphae, conidiophores, and conidia), individual samples of each cell type were run through DESeq2 v.1.50.2 (BiocManager v1.30.27) in R (86) to filter out low reads (<5).

### Flow cytometry analyses

Flow cytometry was performed at the Center for Tropical and Emerging Global Diseases Cytometry Shared Resource Laboratory at the University of Georgia. The Cytek Amnis ImageStreamX Mk II Imaging Flow Cytometer (Cytek Bioscience) was used for all flow cytometry experiments as previously described (87, 88) with slight modifications. Two gates were created using 2–15 µm standards (ThermoFisher Scientific) to filter out non-focused cells and doublets or debris, respectively (Fig. S1B through D). Gates were further optimized for *A. fumigatus* using the IDEAS analysis program (version 6.2). *A. fumigatus* ungerminated conidia (in water) or 5-h germinated conidia (AMM, 37°C) were normalized to 1 × 10^8^ conidia/mL for flow cytometry experiments. Microscope images and data were collected on 10,000 events per sample, and three biological replicates were pooled per sample. The area data in approximate µm^2^ resulting from the approximate 1,500–3,000 events were further analyzed using GraphPad Prism software version 10. Statistical analyses on area or fluorescence data from gated events were performed using Mann-Whitney nonparametric *t*-tests using GraphPad Prism version 10 to compare the distribution among sample groups (two-tailed). *P* values are indicated by stars: ***P < 0.001 and ****P < 0.0001.

## Data Availability

RNA-seq data were submitted to NCBI GEO under BioProject PRJNA1482647.

## SUPPLEMENTAL MATERIAL

Fig. S1 mbio.01831-26-s0001.pdf Growth analysis and flow cytometry.

Fig. S2 mbio.01831-26-s0002.pdf Conidiophore isolation protocol overview.

Fig. S3 mbio.01831-26-s0003.pdf GO enrichment analyses of conidiophore sDEGs.

Fig. S4 mbio.01831-26-s0004.pdf GO enrichment analyses of conidial sDEGs.

Table S1 mbio.01831-26-s0005.xlsx Flow cytometry statistics.

Table S2 mbio.01831-26-s0006.xlsx Total genes expressed in each cell type.

Table S3 mbio.01831-26-s0007.xlsx Differential 37°C vs 50°C.

ASM does not own the copyrights to Supplemental Material that may be linked to, or accessed through, an article. The authors have granted ASM a non-exclusive, world-wide license to publish the Supplemental Material files. Please contact the corresponding author directly for reuse.
