# Supplementary material for: Temperature during conidiophore development primes Aspergillus fumigatus spore transcriptome for asexual or sexual reproduction
Source: mBio. 2026 Sep 1;17(10):e01831-26. doi: 10.1128/mbio.01831-26 (PMC13712828; doi:10.1128/mbio.01831-26)
Supplement: Supplementary file 1 [file mbio.01831-26-s0001.pdf]

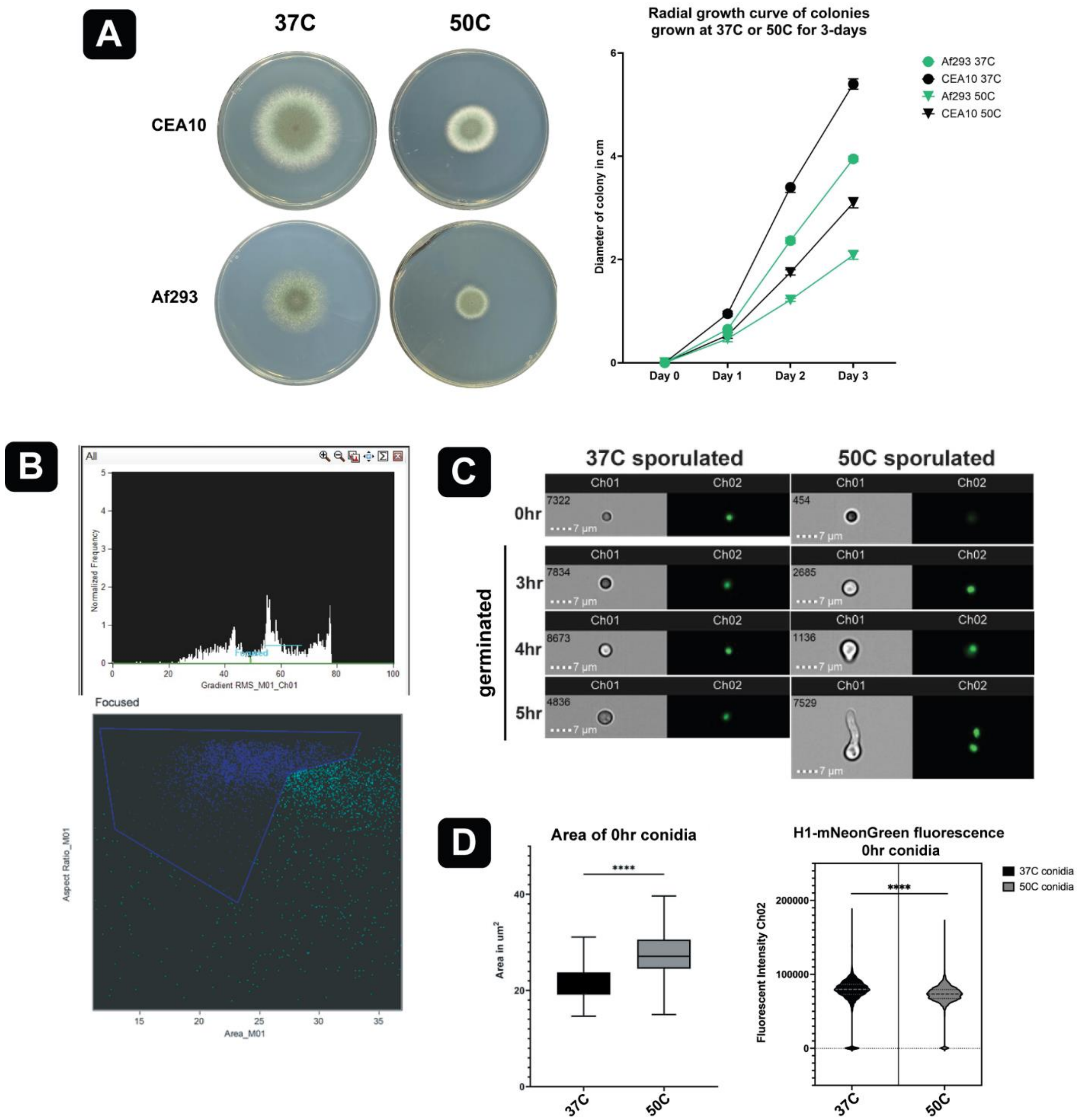

Fig S1. Growth analysis and flow cytometry

(**A**) 3-day colony growth analyses for strains CEA10 and Af293 grown at 37°C (green) and 50°C (black). n=3. **B**-**D**. Flow cytometry experiments utilizing the ImageStream®X Mk II Imaging Flow Cytometer (Cytek® Amnis®). (**B**) Gates created based upon imaging data of the fully detailed events using the IDEAS software program. IDEAS screenshots of flow cytometry gating parameters for *A. fumigatus* conidia (in water). Ch01 shows brightfield, Ch02 displays histone H1-mNeonGreen (nuclear) fluorescence. (**C**) ImageStream images of dormant (0 hour) conidia produced at 37°C (black) or 50°C (grey) or germinated cells after 3-5 hours of incubation at 37°C. Images are representative of the largest, in-focus cells of the population. (**D**) Box plot (left) indicates the area range of dormant conidia produced at 37°C and 50°C (n = 1,500-3,000 events per sample) in μm^2. The middle line represents the median. Mann-Whitney nonparametric test resulting p-value of <0.0001 indicated by stars ****. Violin plots (right) indicate fluorescence intensities of dormant conidia produced at 37°C or 50°C (n = approximately 1,500-3,000 events per sample, Ch02, mNeonGreen). Mann-Whitney nonparametric test resulting p-value of <0.0001 indicated by stars ****.
