# Supplementary material for: Temperature during conidiophore development primes Aspergillus fumigatus spore transcriptome for asexual or sexual reproduction
Source: mBio. 2026 Sep 1;17(10):e01831-26. doi: 10.1128/mbio.01831-26 (PMC13712828; doi:10.1128/mbio.01831-26)
Supplement: Supplementary file 2 [file mbio.01831-26-s0002.pdf]

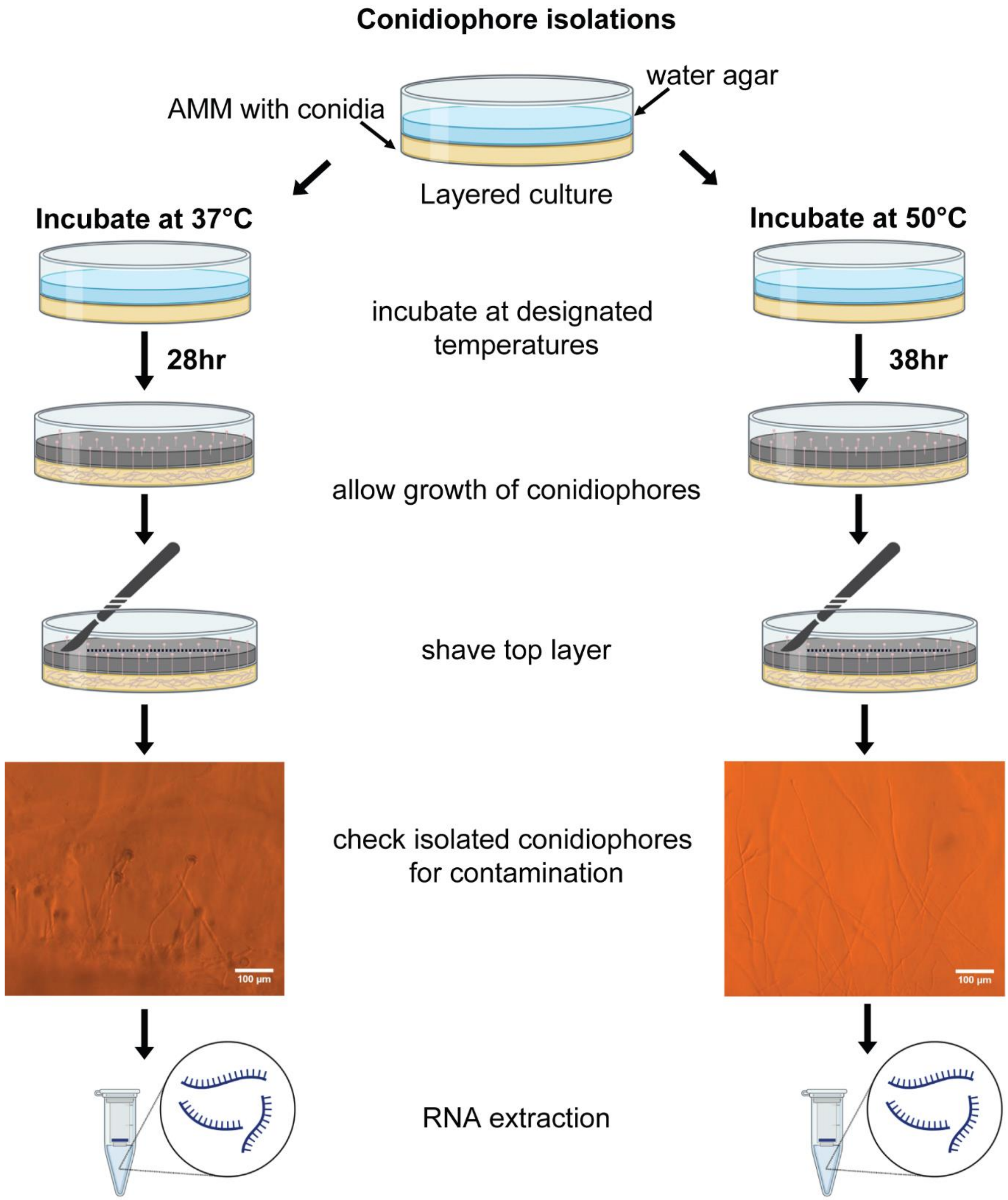

Fig S2. Conidiophore isolation protocol overview. Conidiophores were physically isolated by inoculating a mixture of $2x10^7$ conidia/mL in 40 mL melted AMM, mixed and poured directly into 60 x 15 mm petri dishes. After solidification, 4mL water agar (0.7%) was layered on top and allowed to solidify. Petri plates were incubated at 37°C or 50°C until conidiophores grew through the top agar at the specified time points: 37°C conidiophores were isolated after 28 hours, and 50°C conidiophores were isolated 38 hours after germination. Wet mounts were examined microscopically for each condition to ensure conidiophore purity prior to RNA extractions.
