# Supplementary material for: Temperature during conidiophore development primes Aspergillus fumigatus spore transcriptome for asexual or sexual reproduction
Source: mBio. 2026 Sep 1;17(10):e01831-26. doi: 10.1128/mbio.01831-26 (PMC13712828; doi:10.1128/mbio.01831-26)
Supplement: Supplementary file 4 [file mbio.01831-26-s0004.pdf]

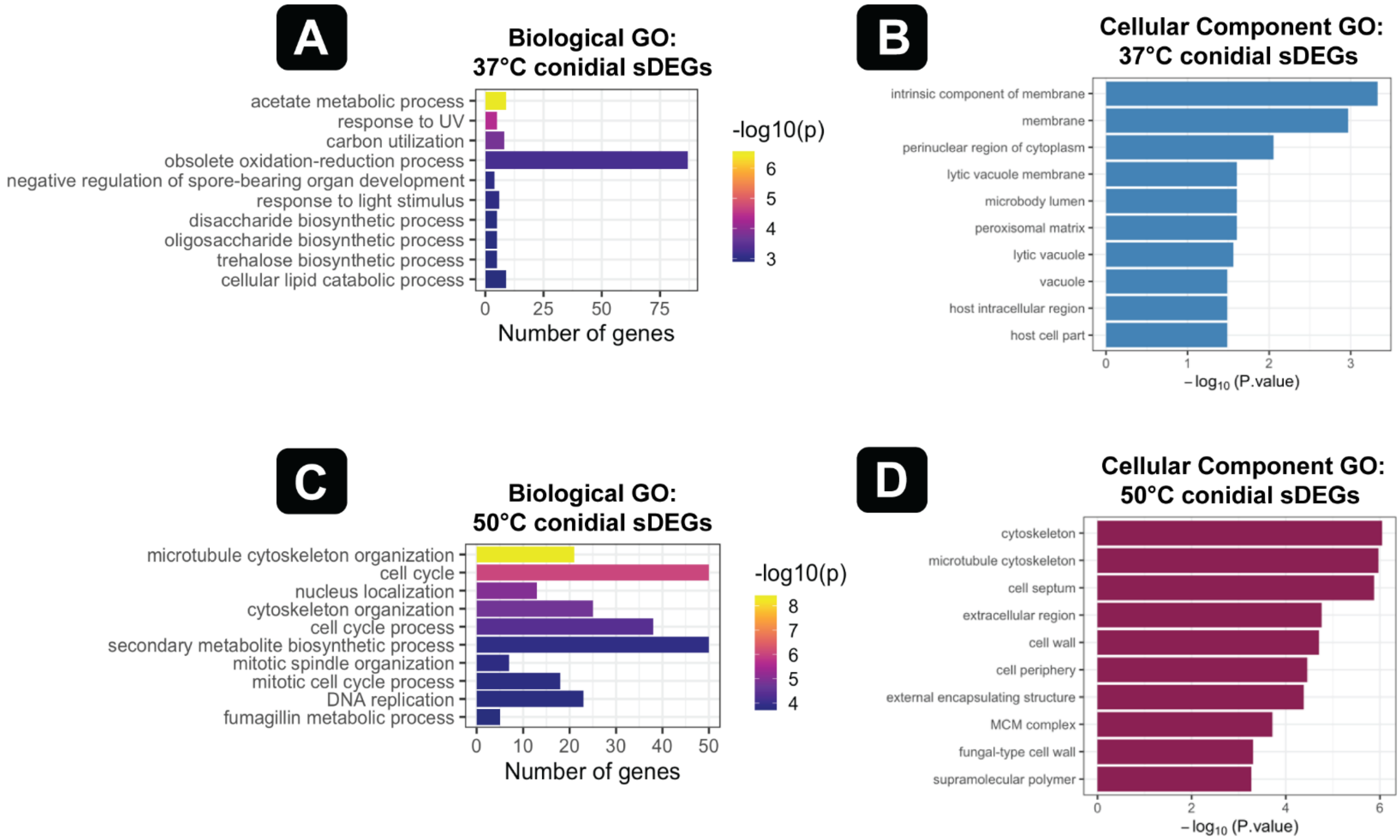

Fig S4. GO enrichment analyses of conidial sDEGs. Biological Gene Ontology (GO) enrichment (**A** and **C**) and Cellular Components GO enrichment (**B** and **D**) from the 37°C versus 50°C conidial differential expression analysis, with any shared upregulated hyphal DEGs removed. GO analyses were carried out using FungiDB (Fisher's exact tests, p-value cutoff of 0.05). Bar graphs display the top 10 most significant GO terms ranked from highest to lowest p-values. (**A**) Biological GO analysis results for 37°C conidial sDEGs (n=972). (**B**) Cellular component GO enrichment for 37°C conidial sDEGs (n=972). (**C**) Biological GO analysis results for 50°C conidial sDEGs (n=1,985). (**D**) Cellular component GO enrichment for 50°C conidial sDEGs (n=1,985).
